# Endogenous anandamide and self-reported pain are significantly reduced after a 2-week multimodal treatment with and without radon therapy in patients with knee osteoarthritis: a pilot study

**DOI:** 10.1007/s00484-021-02095-z

**Published:** 2021-03-01

**Authors:** M. Gaisberger, J. Fuchs, M. Riedl, S. Edtinger, R. Reischl, G. Grasmann, B. Hölzl, F. Landauer, H. Dobias, F. Eckstein, M. Offenbächer, M. Ritter, M. Winklmayr

**Affiliations:** 1 Institute of Physiology and Pathophysiology, Paracelsus Medical University, Strubergasse 21, A-5020 Salzburg, Austria; 2 Gastein Research Institute, Paracelsus Medical University, Salzburg, Austria; 3 Ludwig Boltzmann Institute for Arthritis and Rehabilitation, Paracelsus Medical University, Salzburg, Austria; 4 Dept. of Orthopaedics and Traumatology, Paracelsus Medical University, Salzburg, Austria; 5 Department of Physical Medicine and Rehabilitation, Kardinal Schwarzenberg Klinikum, Schwarzach im Pongau, Austria; 6 Bioanalytical Research Labs, Department of Biosciences, University of Salzburg, Salzburg, Austria; 7 Department of Internal Med., Landesklinik St. Veit im Pongau, SALK, Paracelsus Med. Univ., Salzburg, Austria; 8 Department of Imaging and Functional Musculoskeletal Research, Institute of Anatomy and Cell Biology, Paracelsus Medical University Salzburg and Nuremberg, Salzburg, Austria; 9 Chondrometrics GmbH, Ainring, Germany; 10 Gastein Healing Gallery, Bad Gastein, Austria

**Keywords:** Osteoarthritis, Anandamide, Endocannabinoid, Radon

## Abstract

Multimodal therapies comprising spa applications are widely used as non-pharmaceutical treatment options for musculoskeletal diseases. The purpose of this randomized, controlled, open pilot study was to elucidate the involvement of the endocannabinoid system in a multimodal therapy approach. Twenty-five elderly patients with knee osteoarthritis (OA) received a 2-week spa therapy with or without combination of low-dose radon therapy in the Bad Gastein radon gallery. A 10-point numerical rating scale (pain in motion and at rest), WOMAC questionnaire, and the EuroQol-5D (EQ-5D) questionnaire were recorded at baseline, and during treatment period at weeks one and two, and at 3-month and 6-month follow-ups. Plasma levels of the endocannabinoid anandamide (AEA) were determined at baseline and at 2 weeks, and serum levels of several cartilage metabolism markers at all five time-points. A significant and sustained reduction of self-reported knee pain was observed in the study population, but no further significant effect of the additional radon therapy up and above base therapy. This pain reduction was accompanied by a significant reduction of AEA plasma levels during treatment in both groups. No significant differences were seen in serum marker concentrations between the groups treated with or without radon, but a small reduction of serum cartilage degradation markers was observed during treatment in both groups. This is the first study investigating AEA levels in the context of a non-pharmacological OA treatment. Since the endocannabinoid system represents a potential target for the development of new therapeutics, further studies will have to elucidate its involvement in OA pain.

## Introduction

Osteoarthritis (OA) is a major public health problem among the increasing aged and obese population. OA involves the entire joint and is characterized not only by progressive cartilage breakdown but also by inflammation of the synovial compartment, changes in subchondral bone and osteophyte formation, bone marrow lesions, and changes in the joint capsule and ligaments (Lambova and Muller-Ladner 2018; Man and Mologhianu 2014). According to the WHO, OA is one of the ten most disabling conditions in the developed countries and affects 9.6% of men and 18% of women aged over 60 years worldwide (World Health Organisation 2020). OA pain, the main symptom of OA besides stiffness and joint swelling, severely reduces physical function and health-related quality of life. Besides the individual impairment by chronic pain and reduced joint functionality, OA imposes a high socio-economic burden to public healthcare services, which will further rise due to increasing life expectancy.

There are no disease-modifying drugs (DMOADs) for the treatment of OA available at the moment. Therefore, treatment is restricted to symptoms management like pain reduction, improvement of joint mobility and functionality, and delay of disease progression. Non-steroidal anti-inflammatory drugs (NSAIDs) are commonly used for symptom relief but are also known to elicit potentially serious gastrointestinal and renal adverse effects during long-term oral application (Harirforoosh et al. 2013; Maniar et al. 2018). Often severe and progressive OA ultimately leads to joint replacement surgery. However, between 20 and 30% of patients with hip or knee replacement experience little or no improvement of OA symptoms (Hawker 2019).

Non-pharmacological treatment options for OA include aquatic exercise, gait aids, self-management programs, manual and physical therapy, and balneotherapy (Bannuru et al. 2019; McAlindon et al. 2014; Fitzgerald et al. 2015). Spa therapy comprising mud/peloid packs, thermal or mineral bathes, or natural gases is commonly used in Europe and Middle Eastern countries as a non-pharmacological complementary approach for the treatment of OA or rheumatoid arthritis. Some studies report significant reduction in pain and improvement of health-related quality of life (Antonelli et al. 2018; Fioravanti et al. 2010; Forestier et al. 2010). However, scientific evidence for the efficacy of the different treatment modalities is low (Verhagen et al. 2015).

In Austria the prevalence for OA was estimated to be 11.9% in men and 18.6% in women in 2013 (Dorner and Stein 2013). Thousands of patients mainly from Austria and Germany suffering from OA symptoms utilize the facilities in the Gastein health area which comprise treatments in radon baths and in the radon gallery in addition to individual multimodal treatments. Low-dose radon therapy, i.e., the application of low doses of the noble gas ^222^Rn, is used since decades for the treatment of inflammatory and non-inflammatory degenerative diseases in the Gastein health area as well as in medical spas in Germany, Poland, the Czech Republic, Russia, Japan, France, and many more. Several randomized controlled clinical trials report significant long-term improvement of pain after radon balneology or radon speleotherapy in patients with degenerative spinal disease, rheumatoid arthritis, osteoarthritis, and ankylosing spondylitis (Annegret and Thomas 2013; Falkenbach et al. 2005; Becker 2003; Cuttler 2020; Franke et al. 2007; Kojima et al. 2018; Kuciel-Lewandowska et al. 2020; van Tubergen et al. 2001; van Tubergen and van der Linden 2002). Hormetic effects on DNA repair, the immune system, the antioxidant, or the endocrine system have been proposed; however, the biological mechanisms underlying the potential Rn effects still remain elusive (Galvez et al. 2018; Kuciel-Lewandowska et al. 2018; Lange et al. 2016; Nagy et al. 2009; Shehata et al. 2006; Yamaoka et al. 2005; Ruhle et al. 2019; Ruhle et al. 2017; Wunderlich et al. 2019).

We performed this exploratory, controlled, randomized pilot study to measure the effects of a multimodal treatment with or without additional low-dose Rn therapy on pain, quality of life, and markers of cartilage metabolism. Since the endocannabinoid (EC) system plays an important role in pain modulation and has been implicated in the manifestations of OA pain (La Porta et al. 2015; La Porta et al. 2014), we were also interested in changes of endocannabinoid levels in context of the cure regimen and measured plasma levels of the endocannabinoid anandamide (AEA). Various potential effects of physical therapies including balneotherapy on chronic pain via modulation of the neuroendocrine system have been described previously (Bender et al. 2007; Da Silva and Galdino 2018). Yamaoka et al. reported significantly increased beta-endorphin and ACTH levels in OA patients after a combined hyperthermia and radon inhalation, suggesting that a mitigation of pain by radon treatment might be mediated by β-endorphin (Yamaoka et al. 2004a; Yamaoka et al. 2004b). Interactions and antagonisms of the endogenous opioid system and the EC system have been proposed. They exert their analgesic actions through different but probably interrelated pathways (Bruehl et al. 2019; Crombie et al. 2018; Desroches and Beaulieu 2010; Parolaro et al. 2010; Zubrzycki et al. 2019). To our knowledge, changes in EC levels have not been investigated in the context of OA and physical therapy or radon therapy so far. Results from this study (e.g., effect sizes for sample size calculations) are intended to serve as a basis for future research.

## Material and methods

This longitudinal exploratory pilot study was carried out in the Bad Gastein health area, Austria, and at the Paracelsus Medical University, Salzburg, Austria, between September 2016 and Mai 2017. It was approved by the Salzburg ethics committee (415–E/1965/4–2015). Patients (planned 30) were recruited by a press release and advertisements in local newspapers and included for participation by a physician of the University Hospital for Orthopedics and Traumatology, Salzburg. Inclusion criteria were radiographically diagnosed OA of one or both knees, age 60–75 years, pain score before study onset ≥ 3 on a numeric rating scale (NRS) ranging from 0 (no pain) to 10 (worst possible pain), and suffering for a minimum of 1 year from OA-related pain. Excluded were patients with diseases representing a contraindication to radon therapy in the radon gallery. The recruiting orthopedist also performed the Kellgren-Lawrence classification on the basis of the radiographs.

Enrolled patients were randomized in two arms into a radon and a control group with an online randomization tool (https://www.evidat.com/rita). All study participants stayed in a Bad Gastein health resort (1.000 m a.s.l.) for 2 weeks and received the same number of basic therapies: 4x under water therapies, 4x massage, 2x sensorimotor training, 2x mudpacks, 4x ergometer training, and 2x knee-specific training. The radon group undertook 8 additional 1-h treatments in the radon gallery (yearly average 44 kBq/m^3^). Blood (serum and plasma) and urine samples were collected at start (T0), after 1 week (T1), and at the end of the cure regimen (T2). Follow-ups took place at the Paracelsus Medical University 3 (T3) and 6 (T4) months after the therapy. At all time-points, Western Ontario and McMaster Universities Osteoarthritis Index (WOMAC) questionnaire, the EuroQol-5D (EQ-5D) questionnaire, and a numeric rating scale (NRS) addressing pain in motion and at rest were handed out to the patients for the acquisition of patient-reported outcome. In addition, at the end of the cure regimen (T2), patients completed a short questionnaire addressing their estimation of the treatment success.

### Collection of samples and quantification of biomarkers

Forearm venous blood from all patients was collected at all time-points (T0–T4). Twelve milliliters of blood from each individual was collected in serum and plasma tubes (BD Vacutainer®, Heidelberg, Germany) according to manufacturer’s guidelines, and plasma and serum aliquots were immediately frozen and stored at −80°C until further processing. Urine samples of each participant were equally frozen immediately. All samples were collected in a non-fasted state between 9 and 12 am.

Serum levels of cartilage oligomeric matrix protein (COMP) and N-propeptide of collagen IIA (PIIANP) were quantified by enzyme-linked immunosorbent assays (ELISA) according to the manufacturer’s guidelines (COMP: Biovendor, Brno, Czech Republic; PIIANP: Merck, Darmstadt, Germany). Urinary C-terminal cross-linked telopeptide of type II collagen (uCTXII) was analyzed by ELISA (MyBiosource, San Diego, USA), and the raw values normalized to urinary creatinine quantified by diagnostic test strips according to the manufacturer’s protocol (Reflotron® Creatinine, Roche Diagnostics GmbH, Mannheim, Germany)

#### Anandamide quantification

Plasma samples were thawed just prior to sample preparation. Cannabinoid enrichment was performed by solid phase extraction. Thermo 1mL C_18_ HyperSep–Retain SPE cartridges (1 mL, 30 mg column bed) were used to enrich unpolar analytes from plasma samples. After equilibration with 1 mL acetonitrile (ACN; 0.1% formic acid (FA)) and washing with 1 mL H_2_O (0.1% FA) 500μL of plasma spiked with 1 μL of internal standard d_4_-AEA (c = 1 ng mL^−1^) were directly applied onto the column bed, sucked through, washed with 1 mL of 20% ACN (0.1% FA) and sucking through air for 30 s to dry the column bed. Elution of the enriched analytes was done by sucking through 400 μL of ACN (0.1% FA) and directly collecting the flow through in 0.5 mL Eppendorf vials. HPLC-MS measurements were done with a Thermo Ultimate 3000 System, equipped with two different Thermo Hypersil Gold C_18_ columns, a narrow bore 100 mm × 1 mm i.d. column, and a 100 mm × 2.1 mm i.d. column. The complete series was measured on the 2.1 mm i.d. column at a flow rate of 300 μL min^−1^. Injection volume was 5 μL. Mass spectrometric detection was done by selected reaction monitoring using the mass of the intact molecule and the 3 most intensive MS transitions (348.2897 ➔ 287.35, 245.23, and 62.06 m/z). In order to compensate for system performance fluctuations, MS ion suppression effects and varying losses through sample preparation analyte peak areas were normalized to the internal standard peak area.

### Statistical analysis

Statistical analyses were performed using SPSS software (SPSS Inc., Chicago, IL, USA, version 24), and graphs were created using GraphPad Prism 8.1.2 (GraphPad Software, San Diego, CA, USA). All variables are expressed as mean ± standard deviation (SD) unless otherwise indicated. A linear mixed model approach with time, treatment, and time*treatment as fixed factors was used for statistical analysis. Statistical significance was set at the level of *α* ≤ 0.05 for all tests.

## Results

### Study participants and baseline characteristics

**Fig. 1:**
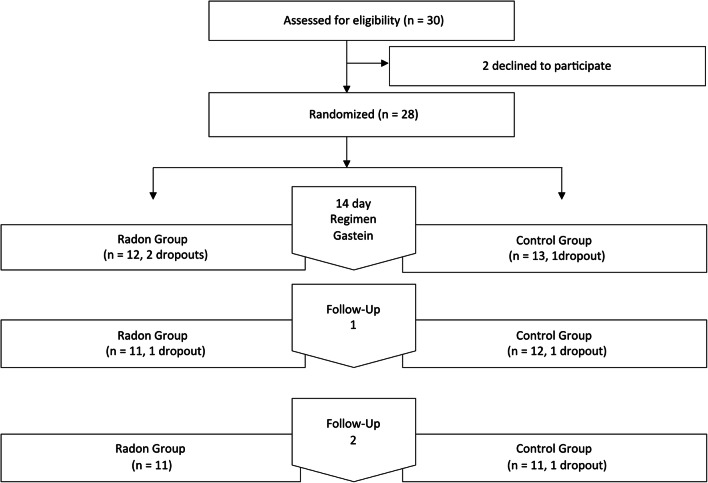
Study flow chart

Figure 1 shows the number of patients at each stage of the study. Of 30 enrolled participants, 28 were randomized into the two study arms. There were three dropouts during the cure regimen, which were excluded from the statistical analysis and another three dropouts during the follow-ups. Since questionnaires could be obtained from the latter ones by mail, they remained in the analysis. Baseline characteristics do not show any statistically relevant differences between the radon and the control group at the start of the study (Table 1).

**Table 1:** Baseline statistics

	Radon	Control
Number	13	12
Female	7	8
Male	6	4
Age	67.23 ± 4.75	67.50 ± 3.99
BMI	26.45 ± 4.93	27.99 ± 4.28
Kellgren grade	2.67 ± 0.65	2.58 ± 0.69
WOMAC total score	33.65 ± 17.28	32.33 ± 14.98
WOMAC pain	36.77 ± 14.20	34.83 ± 14.83
WOMAC stiffness	35.00 ± 21.21	43.75 ± 22.48
WOMAC physical	32.58 ± 20.30	30.25 ± 16.66
EQ-5D score	0.77 ± 0.10	0.77 ± 0.10
Pain in potion (NRS)	5.00 ± 1.71	5.25 ± 2.17
Pain at rest (NRS)	3.42 ± 1.40	3.21 ± 1.75
COMP ng/mL	34.14 ± 24.71	27.27 ± 8.33
PIIANP ng/mL	586.69 ± 110.45	705.24 ± 199.75
uCTXII ng/mMol Crt	13.56 ± 6.21	15.05 ± 12.75

### Assessment of OA (WOMAC), pain (NRS), and health-related quality of life (EQ-5D)

**Fig. 2:**
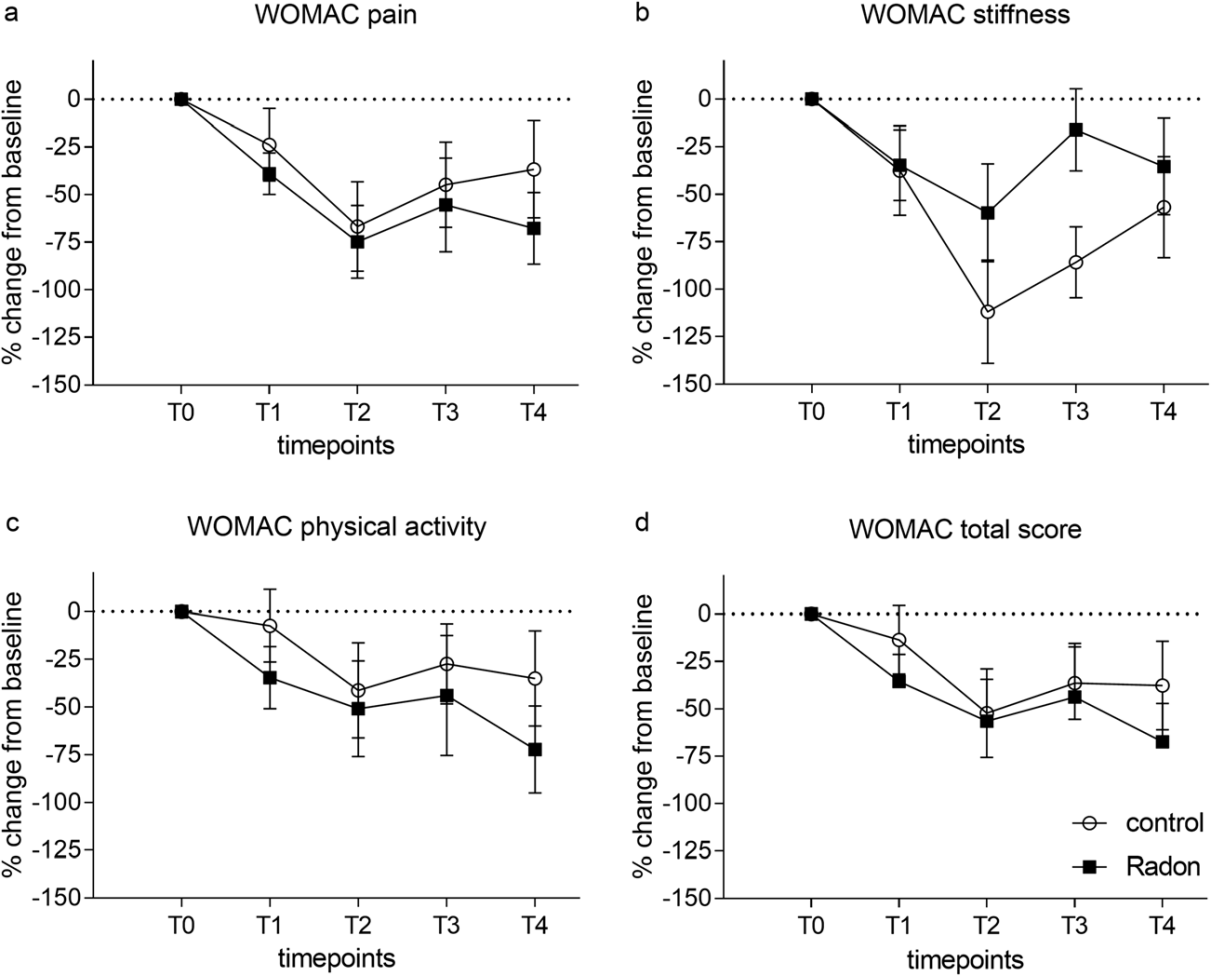
Time course of changes in WOMAC scores relative to baseline values. **a** WOMAC pain, **b** WOMAC stiffness, **c** WOMAC physical activity, and **d** WOMAC total. Black rectangles, radon group; circles, control group. Mean ± SEM

The Western Ontario and McMaster Universities Arthritis Index (WOMAC) is internationally used for the assessment of knee and hip OA. Figure 2 shows the course of the WOMAC total score (a) and the three subscales pain (b), stiffness (c), and physical function (d) during the cure regimen (T0-T2) and the follow-ups (T3–T4). Statistical analysis revealed a significant overall reduction of all scores over time (pain: *p* < 0.001; stiffness: *p* < 0.001; physical activity: *p* = 0.026; total score: *p* = 0.002). However, between-group pairwise comparisons did not disclose any significant impact of the radon treatment over time. The reduction of all scores is most prominent at T2 at the end of the patient’s sojourn in Bad Gastein, and then the scores rise until the last measurement but are still below starting values indicating a sustained improvement of OA symptoms even 6 months after the end of the cure regimen.

**Fig. 3:**
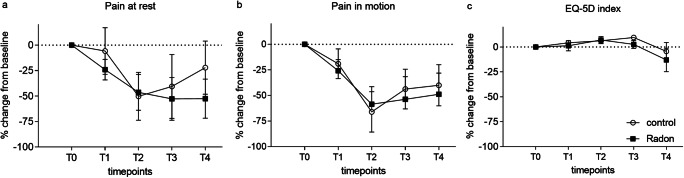
Time course of changes in pain (NRS) (**a** and **b**) and EQ-5D scores (**c**) relative to baseline values. Black rectangles, radon group; circles, control group. Mean ± SEM

The reduction of WOMAC OA scores is also reflected by a mitigation of self-assessed pain in a numeric rating scale ranging from 0 (no pain at all) to 10 (worst imaginable pain). Pain at rest (Fig. 3a) as well as pain in motion (Fig. 3b) is significantly ameliorated in the whole study population over time (pain at rest: *p* = 0.005; pain in motion: *p* = 0.001), showing up to 50% improvement of pain at the end of the treatment phase. However, additional radon therapy again did not cause any further statistically relevant improvement compared to the control group. Pain-reducing effects of the whole treatment are still evident after 3 and 6 months. In contrast, health-related quality of life assessed by the EQ-5D questionnaire is slightly but not statistically significant elevated until T2 in both groups and then drops below baseline values at T4.

### Evaluation of clinically relevant changes

**Fig. 4:**
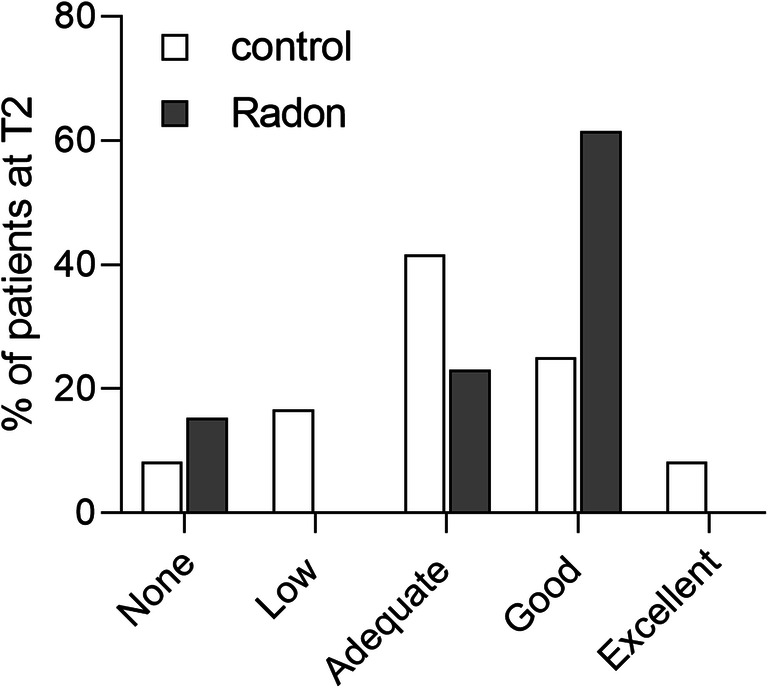
Self-estimated treatment effect at T2

To interpret changes in OA scores at the individual level, we measured the portion of improved patients at the end of the treatment phase by a short questionnaire addressing the self-estimated success of the therapy on a 5-point Likert scale (1, no effect at all; 2, small effect; 3, adequate effect; 4, good effect; 5, excellent effect). 61.54% of patients in the radon group assessed a good effect of the treatment at T3, compared to 25% in the control group (Fig. 4). In the latter, more patients experienced an adequate improvement than in the radon group (control: 41.67%; radon: 32%). This putative advantage of the radon treatment has, of course, to be considered cautiously, since the treatment in the radon gallery cannot be blinded, and the perceived improvement might be caused by an additional placebo effect.

The minimal clinically important improvement (MCII) is defined as the smallest change in measurement that signifies an important improvement. Among the patients, who considered the effect of the radon treatment as good, 75% experienced a decrease in pain > 2 points on a 10-point NRS for pain in motion.

### Anandamide levels

**Fig. 5:**
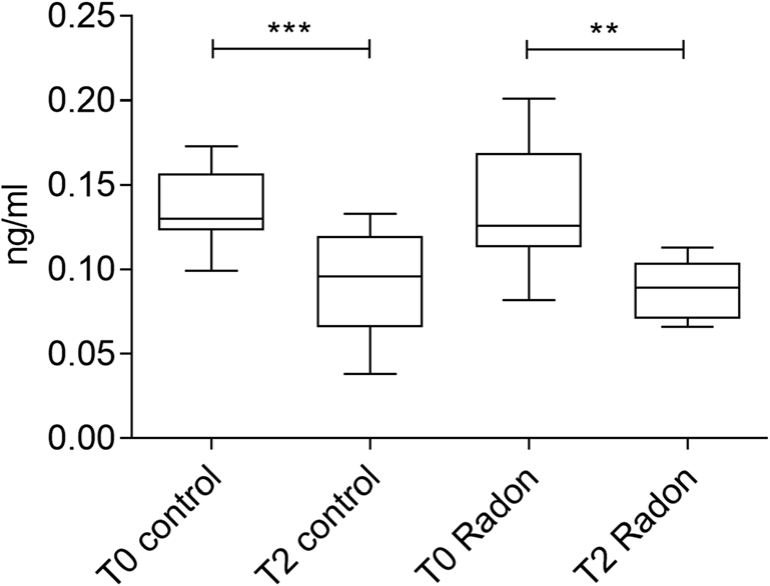
Plasma levels of AEA in ng/mL (*n*=22); **p≤0.01; ***p≤0.001

The question, whether the known pain reducing effects of the Gastein multimodal therapy affect the endocannabinoid system or are mediated by it, prompted us to quantify the endogenous cannabinoid anandamide (AEA) in the plasma samples. We were able to determine AEA concentrations in 22 samples, and in 3 samples, AEA levels were beyond the detection limit. Analysis revealed a significant reduction of AEA after the 2-week cure regimen in both treatment groups (Fig. 5).

### Quantification of biochemical markers of cartilage metabolism

Levels of the putative cartilage degradation markers urinary CTXII and serum COMP and serum levels of PIIANP are shown in Table 2. Serum COMP levels decline in both groups to a minimum at T3 (*p* = 0.041) indicating a potential chondroprotective effect of the whole treatment, but no additional effect of the radon therapy. This is supported by a slight but not statistically relevant decline in uCTX levels in both groups during the cure regimen. Serum PIIANP levels, indicative of type II collagen synthesis, reveal individual variations, but in sum remain unaffected by the treatment.

**Table 2:** Concentrations of biomarkers

	PIIANP		uCTXII		COMP	
	Control	Radon	Control	Radon	Control	Radon
T0	693.00 ± 217.71	599.03 ± 118.65	16.01 ± 12.90	12.10 ± 5.56	26.27 ± 7.21	34.63 ± 28.51
T1	538.79 ± 248.87	565.58 ± 190.85	10.19 ± 12.74	6.58 ± 3.85	27.90 ± 11.22	29.56 ± 14.10
T2	626.57 ± 229.62	594.58 ± 178.48	15.41 ± 27.23	6.12 ± 3.64	26.78 ± 7.93	28.93 ± 17.37
T3	677.35 ± 226.34	669.38 ± 190.57	10.97 ± 10.96	13.88 ± 11.71	21.63 ± 4.24	24.70 ± 7.51
T4	657.25 ± 232.09	613.42 ± 146.50	10.54 ± 9.32	13.71 ± 15.45	22.05 ± 5.39	28.48 ± 18.13

Serum PIIANP ng/mL and serum COMP ng/mL; urinary CTXII ng/mM creatinine. Mean ± SD

## Discussion

In the present explanatory, longitudinal pilot study with 25 OA patients, we investigated the effects of a multimodal treatment and additional radon therapy on pain and biomarkers of cartilage metabolism. In addition, we quantified levels of endogenous AEA to determine potential effects of the cure regimen on the endocannabinoid system.

Statistical analysis revealed a significant sustained reduction of the WOMAC total score and the WOMAC subscores for pain, stiffness, and physical function. A significant reduction of pain was also evident in pain numeric rating scales addressing pain in motion and pain at rest. Although symptom reduction seemed slightly stronger and more sustained in the radon group (WOMAC pain, physical activity, total; NRS), no statistically significant impact of the additional radon treatment could be shown in this pilot study. Health-related quality of life was improved as well in both groups until 3 months after the end of the cure regimen.

Interestingly, we could also show a significant decline of AEA plasma levels during the cure regimen in both groups. The endocannabinoid AEA has been shown to modulate inflammation by suppressing the production of pro-inflammatory cytokines. It also inhibits NO synthesis and TNF-α-mediated activation of transcription nuclear factor B (Donvito et al. 2018). Clinical studies have demonstrated that CB1 and CB2 receptor mRNA and proteins are expressed in the synovia of OA and RA patients and that the endocannabinoids 2-AG and AEA are present in the synovial fluid of these patients, but not in the synovial fluid of individuals without joint symptoms (Richardson et al. 2008), suggesting an upregulation of the EC system to counteract inflammation and cartilage degradation in OA patients. The downregulation of AEA levels in our study population in combination with the reduction of pain symptoms might therefore indicate an important influence of the cure regimen on the regulation of inflammation and pain mediating processes. Since the EC system is associated with inflammatory, neuropathic, and OA pain, it represents a promising target for the development of new therapeutics. The present study is to our knowledge the first examining changes of AEA levels in OA patients during a cure regimen.

The value of biomarkers in OA research is presently a focus for intensive discussion (Lotz et al. 2014; van Spil and Szilagyi 2020). Collagen II degradation marker uCTXII together with sCOMP, also associated with cartilage degradation, are the most frequently investigated markers in OA. Elevated levels of uCTXII have been associated with radiographic progression of OA (Dam et al. 2009). Lower PIIANP has been shown to be associated with a greater radiographic disease burden in knee and hip joints (Daghestani et al. 2017). The measurement of COMP, PIIANP, and UCTXII in blood and urine was aimed to identify potential biological markers of cartilage metabolism responding to the intervention and to investigate potential effects of the treatment on cartilage homeostasis. We observed a reduction of COMP and uCTXII levels during the cure regimen in both groups, whereas PIIANP levels stayed more or less unaffected over time.

In summary we could show a significant and sustained reduction of pain and AEA levels in the whole study population, but no additional beneficial effect of the Rn treatment. More study participants in the radon group than in the control group considered the effect of the cure regimen as good, but as mentioned above, this might be due to the not blinded design of the study. Due to high costs arising from accommodation and meals offered to the participants in addition to therapeutic interventions, the sample size in our pilot study was quite small. High individual variability in all outcome variables led to very high standard deviations and did not provide sufficient statistical power to detect a difference between the control group and the group receiving additional radon treatments. A posterior sample size calculation revealed that 568 patients would have been required to have a 80% chance of detecting a significant change at the 5% level in the primary outcome (WOMAC total score T4) measure.

Many effects of radon therapy have been shown for systemic inflammatory diseases (Falkenbach et al. 2005; Lange et al. 2016; Shehata et al. 2006; van Tubergen et al. 2001). OA is primarily considered a non-inflammatory degenerative disease, although the influence of inflammatory components is more and more recognized, and radon therapy might simply be without apparent measurable effect in the observed variables in OA patients. A further limitation is also represented by the fact that our study participants received a smaller amount of Rn applications in a shorter time course (8 treatments in 2 weeks) compared to 12 and more treatments in a regular cure regimen lasting up to 4 weeks. Potential small effects of the Rn treatment might be masked by the overall cure effect of the multimodal treatment, by the change of climatic or environmental conditions or simply by a holiday effect.

Balneotherapy or spa therapy is often prescribed as complementary therapy, but also for patients suffering from severe side effects by pharmacological treatments or from comorbidities contraindicating pharmacological treatment. However, scientific evidence for these treatments is low. Our study was aimed to provide a basis for further and larger studies about the impact of complementary therapies for OA.

## Data Availability

Not applicable
